# Hybrid Satellite-Terrestrial Relay Network: Proposed Model and Application of Power Splitting Multiple Access

**DOI:** 10.3390/s20154296

**Published:** 2020-08-01

**Authors:** Dinh-Thuan Do, Anh-Tu Le, Rupak Kharel, Adão Silva, Mohammad Abu Shattal

**Affiliations:** 1 Department of Computer Science and Information Engineering, College of Information and Electrical Engineering, Asia University, Taichung City 41354, Taiwan; 2 Faculty of Electronics Technology, Industrial University of Ho Chi Minh City (IUH), Ho Chi Minh City 700000, Vietnam; leanhtu@iuh.edu.vn; 3 Department of Computing and Mathematics, Manchester Metropolitan University, Manchester M15 6BH, UK; 4 Instituto de Telecomunicações (IT) and Departamento de Eletrónica, Telecomunicações e Informática (DETI), University of Aveiro, 3810-193 Aveiro, Portugal; asilva@av.it.pt; 5 Department of Computer Science and Engineering, The Ohio State University, 2015 Neil Avenue, Columbus, OH 43212, USA; abushattal@ieee.org

**Keywords:** energy harvesting, ergodic capacity, NOMA, outage probability, satellite communications

## Abstract

The development of hybrid satellite-terrestrial relay networks (HSTRNs) is one of the driving forces for revolutionizing satellite communications in the modern era. Although there are many unique features of conventional satellite networks, their evolution pace is much slower than the terrestrial wireless networks. As a result, it is becoming more important to use HSTRNs for the seamless integration of terrestrial cellular and satellite communications. With this intent, this paper provides a comprehensive performance evaluation of HSTRNs employing non-orthogonal multiple access technique. The terrestrial relay is considered to be wireless-powered and harvests energy from the radio signal of the satellite. For the sake of comparison, both amplify-and-forward (AF) and decode-and-forward (DF) relaying protocols are considered. Subsequently, the closed-form expressions of outage probabilities and ergodic capacities are derived for each relaying protocol. Extensive simulations are performed to verify the accuracy of the obtained closed-form expressions. The results provided in this work characterize the outage and capacity performance of such a HSTRN.

## 1. Introduction

Satellite communication is considered to be one of the most reliable forms of wireless communications. Satellite communication networks have several advantages over conventional wireless communications that include long-distance communication, large coverage area, and flexible communication environments [1]. Due to this, their role in emergency applications is growing each passing year. This is especially true in a situation where the communication infrastructure is destroyed due to large-scale disasters [2]. Even when a few communication access points are available in the disaster-ridden region, there is the possibility of network congestion and overload due to increased demand. This makes satellite communication more feasible and important to carry out disaster relief operations [3].

Although mobile satellite networks can prove advantageous in disaster management, the issues of operation costs and transmission capacity cannot be ignored in such networks. Thus, it is important to establish a flexible, stable, and broadband communication network in disaster regions [4]. Furthermore, when the terrestrial user is not outdoor or the angles of satellite elevation are low, the conventional mobile satellite networks suffer crucial performance degradation. In this context, different relaying techniques can be employed to improve the coverage and reliability of the network. This form of networks are generally called hybrid satellite-terrestrial relay network (HSTRN) and have shown to significantly decrease the masking effect [5,6]. The HSTRNs can integrate the advantages of both conventional satellite networks and the terrestrial broadband networks. Broadly speaking, they have the ability to provide longer transmission coverage along with higher data rates without environmental limitations. These hybrid networks are able to provide multimedia services while ensuring the quality of service requirements for mobile users through ITU-R S.2222 standard cross-layer design [7]. The HSTRNs are, thus, considered to be one of the promising solutions for ensuring public safety in disaster regions due to their performance gains.

In parallel, non-orthogonal multiple access (NOMA) techniques have received considerable research interest due to their ability to improve the spectral efficiency of wireless networks [8]. For instance, using NOMA can be combined with cognitive radio to increase spectrum efficiency [9]. Although the applications of NOMA in HSTRNs have not been explored extensively, significant performance improvements can be achieved with NOMA-enabled HSTRNs. NOMA also improves user fairness, thereby, allow different users to experience the same quality of services. NOMA also does not require significant modifications in the existing network architecture [10], thus, operating with virtually no change in the cost of deployment and operation of the network. These exciting benefits of NOMA-enabled HSTRNs motivate us to further explore the performance limits of such networks and identify the performance variations under different network parameters. In the following, we outline some of the recent state-of-the-art developments in this domain.

### 1.1. Related Work

Regarding developing applications of satellite communication systems, there was considerable attention paid to the research community since these systems are widely implemented in the field of broadcasting and navigation for their larger coverage in serving terrestrial devices [11,12]. Since obstacles exist in the link from terrestrial users to satellite and it limits the transmission of the line of sight (LoS). As a result, the masking effect occurs in such systems as the main impairment. In recent technical literature, the HSTRN has been extensively investigated. For example, in [13,14,15], amplify-and-forward (AF) relaying is employed to enhance the performance of HSTRN. In contrast, HSTRN was considered in relaying mode using the decode-and-forward (DF) as in [16,17]. The HSTRN was studied in a system model that combines the beamforming technique and AF transmission mode [18]. The author in [19] studied an overlay cognitive hybrid satellite–terrestrial network allowing a primary satellite source–receiver pair and a secondary transmitter–receiver pair can operate under impact of practical hardware impairments (HIs). The DF-based secure 3D mobile unmanned aerial vehicle (UAV) relaying was investigated in the scenario of satellite-terrestrial networks [20]. In this case, probability of non-zero secrecy capacity (PNZSC) and secrecy outage probability (SOP) are presented. In the context of all network nodes are subjected to hardware impairments, a multi-relay selection (MRS) scheme is studied to improve the outage performance of the HSTRN [21].

Recently, to improve spectral efficiency NOMA was introduced as one promising architecture for multiple access applied in future fifth-generation (5G) wireless networks [22]. By permitting multiple users to be served by same resource (the same time, frequency or code domain), the improved spectral efficiency and user fairness are provided in NOMA, which is different compared with traditional orthogonal multiple access (OMA) In particular, the transmitter experiences superposing signal from multiple users while successive interference cancellation (SIC) is implemented at the receivers which are required to separate the mixture signals in the power domain [23]. Specifically, in [24], a cooperative network and NOMA are employed to form the NOMA (C-NOMA) scheme and then this scheme is facilitated in NOMA-based cellular network with multiple users. In their system, relays are adopted to assist the users with weak channel conditions while users with strong channel conditions acted as such relay. In [25], ergodic capacity and outage probability were determined to measure the quality of NOMA transmission in a cellular system.

However, a limited energy situation exists in the relay node in this C-NOMA. It is hard to replace the battery and/or there is no power line. The energy from the surrounding environments can be reused to address this problem, i.e., via an energy harvesting technique. Regarding radio frequency (RF) energy harvesting [26], stable energy supply can be achieved and such an energy harvesting scheme has been widely studied in cooperative networks in which relay harvests the energy from the radio-frequency signals [27,28]. An HSTRN using the NOMA scheme was proposed [29], in which a user with better channel condition is adopted as a relay node and forwarded the information to other users. The satellite in the downlink is considered in the scenario in which a relay node is employed to retransmit the NOMA signal from the satellite [30]. To validate the effectiveness of the proposed system model, and the closed-form outage probability expressions are derived. However, works [29,30] mainly conducted performance evaluation based on a fixed power source at the relay, without considering energy harvesting strategy in a scenario where relay can harvest energy from the satellite directly.

### 1.2. Motivation and Contribution

From the aforementioned analysis, one can deduce that there is a serious lack of understanding of different aspects of energy harvesting HSTRNs that employ NOMA. To fill this research gap as well as meet the requirements of both performance and energy efficiency (EE) in future satellite communications, in this paper, we provide an in-depth performance evaluation of NOMA-enabled HSTRNs that employ energy harvesting relays. Our key contributions can be summarized as follows:

1. A dynamic multi-antenna satellite-terrestrial relay communication design was considered. The terrestrial relay is able to harvest energy from the RF signal from the satellite and use it to forward the message to users. As main kind of NOMA, i.e., power splitting multiple access, is studied. The wireless channel between relay and NOMA users experiences Nakagami-*m* fading which is more versatile than conventionally used Rayleigh fading.
2. The closed-form expressions of outage probabilities of AF and DF relays are provided once Shadowed-Rician fading model is applied for satellite link. In addition to this, the analytical expressions of ergodic capacities are provided.
3. Extensive simulations are carried out to validate the accuracy of derived expression. The obtained results are also compared with the conventional OMA approach to highlight the performance gains.

### 1.3. Organization

The remainder of the paper is organized as follows. Section 2 provides details of the system model. In Section 3, outage probability and ergodic capacity expressions are provided. Section 4 presents numerical results and discussion. Finally, Section 5 provides some future research directions along with concluding remarks.

## 2. System Model

**Figure 1:**
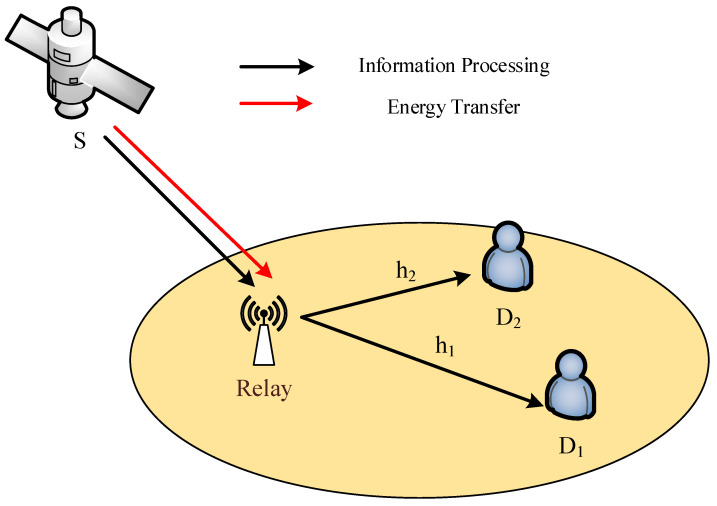
Illustration of energy harvesting HSTRN using NOMA.

Let us consider a land-mobile satellite communication system with a relay and two terrestrial users $D_{1},D_{2}$ as Figure 1. The relay are equipped with a single antenna while the satellite and terrestrial users are equipped with *M* and $N_{i}$ antennas, respectively. The key parameters are described in Table 1. The ground users employ the power-domain NOMA technique and decode the superimposed signal transmitted by satellite. The wireless link from terrestrial relay to $D_{i}$, is denoted by $h_{i}$. The channel from the relay to users is assumed to undergo Nakagami-*m* fading with fading severity $m_{i}$, average power $\Omega_{i}$ and channel gains are $\Lambda_{i}=\frac{\Omega_{i}}{m_{i}}$. Furthermore, the satellite links are experienced by Shadowed-Rician fading [31]. It is further assumed that the receiving nodes are distributed as additive white Gaussian noise (AWGN) with mean zero and variance $N_{0}$. The overall communication operates in two orthogonal time slots and it is considered that no direct link exists between the satellite and the NOMA users. During the first time slot, the satellite sends the superimposed signal to the terrestrial relay. The terrestrial relay deploys the time switching-based relay energy harvesting protocol to harvest energy from the radio signal of the satellite. During the second time slot, the NOMA users decode their individual signals with the help of SIC. For the sake of detailed performance evaluation, we consider AF and DF relaying protocols. The working of these relaying protocols in the considered system model is elaborated in following paragraphs.

### 2.1. DF Protocol

In case of DF relaying protocol, relay first decodes the data and then forwards it the the users. With the aid of an energy harvesting-enabled relay, the end-to- end communication occurs in two time phases. To save energy, the relay uses only single antenna to receive the signal from satellite. It then uses *N* antennas for maximum ratio transmission (MRT) to transmit the signal to NOMA users [32]. Considering the data transmission at the first sub-block in which the multiple antennas satellite S transmits an superimposed information signal $x=\sqrt{\Xi_{1}}x_{1}+\sqrt{\Xi_{2}}x_{2}$. In the first phase

(1) $$y_{R}=\sqrt{P_{S}}h_{SR}^{†}w_{SR}\left(\sqrt{\Xi_{1}}x_{1}+\sqrt{\Xi_{2}}x_{2}\right)+n_{R},$$

where $h_{SR}^{}={\left[h_{SR}^{1}\cdots h_{SR}^{M}\right]}^{T}$ is the M×1 channel vector from S to R. For MRT, $w_{SR}=\frac{h_{SR}^{}}{{∥h_{SR}^{}∥}_{F}}$, where ${∥.∥}_{F}$ is Frobenius norm and † denotes conjugate transpose. Following decoding of NOMA, signal decoding order is decided based on order of channel gains. We assume that $\Xi_{1}>\Xi_{2}$. Then, the signal- to-interference-and-noise ratio (SINR) to detect $x_{1}$ can be given as

(2) $$\Gamma_{R\to x_{1}}^{DF}=\frac{{\bar{\gamma }}_{SR}\Xi_{1}}{{\bar{\gamma }}_{SR}\Xi_{2}+1},$$

where $\rho_{S}=\frac{P_{S}}{N_{0}}$ and ${\bar{\gamma }}_{SR}=\rho_{S}{∥h_{SR}∥}_{F}^{2}$. After performing SIC at relay the signal-to-noise ratio (SNR) to detect $x_{2}$ can be formulated by

(3) $$\Gamma_{R\to x_{2}}^{DF}={\bar{\gamma }}_{SR}\Xi_{2}.$$

Next, energy harvesting is employed at the relay and energy level achieved at relay is given by [33]

(4) $$E_{R}=\eta P_{S}{∥h_{SR}∥}_{F}^{2}\chi T.$$

From this point, it can be computed transmit power at the relay as

(5) PR=ER1−χTT22=2ηPShSRF2χ(1−χ).

In the second phase, the MRT is used with beamforming vector such that we obtain $w_{i}=\frac{h_{i}}{∥h_{i}∥}$ in which $∥.∥$ denotes the Euclidean norm of a matrix. Then, the received signal achieved at user $D_{i}$ is given by

(6) $$y_{D_{i}}^{DF}=P_{R}∥h_{i}w_{i}∥\left(\sqrt{\Xi_{1}}x_{1}+\sqrt{\Xi_{2}}x_{2}\right)+n_{D_{i}}$$

The SINR at $D_{1}$ to decode $x_{1}$ can be expressed by

(7) $$\Gamma_{D_{1}\to x_{1}}^{DF}=\frac{P_{R}{∥h_{1}∥}^{2}\Xi_{1}}{P_{R}{∥h_{1}∥}^{2}\Xi_{2}+N_{0}}=\frac{\phi {\bar{\gamma }}_{SR}{∥h_{1}∥}^{2}\Xi_{1}}{\phi {\bar{\gamma }}_{SR}{∥h_{SR}∥}^{2}\Xi_{2}+1},$$

where $\phi =\frac{2\eta \chi }{\left(1-\chi \right)}$.

In a similar way, before and after SIC performed at user $D_{2}$, SNR computation related to detect signal $x_{1},x_{2}$ are respectively given by

(8) $$\Gamma_{D_{2}\to x_{1}}^{DF}=\frac{\phi {\bar{\gamma }}_{SR}{∥h_{2}∥}^{2}\Xi_{1}}{\phi {\bar{\gamma }}_{SR}{∥h_{2}∥}^{2}\Xi_{2}+1},\;\Gamma_{D_{2}\to x_{2}}^{DF}=\phi {\bar{\gamma }}_{SR}{∥h_{2}∥}^{2}\Xi_{2}.$$

### 2.2. AF Protocol

The AF relaying protocols amplifies the received signal before transmitting it to the users. In this regard, the variable gain is defined as $G=\sqrt{\frac{P_{R}}{{∥h_{SR}∥}_{F}^{2}P_{S}+N_{0}}}$. In the second phase, with the help of Equation (1) the received signal can be expressed as

(9) $$\begin{array}{ll} y_{D_{i}}^{AF} & =∥h_{i}w_{i}∥Gy_{R}+n_{D_{i}}\\ \; & =∥h_{i}w_{i}∥G\sqrt{P_{S}}h_{SR}^{†}w_{SR}\left(\sqrt{\Xi_{1}}x_{1}+\sqrt{\Xi_{2}}x_{2}\right)\\ \; & +∥h_{i}w_{i}∥Gn_{R}+n_{D_{i}}. \end{array}$$

The instantaneous received SINR for $D_{1}$ then becomes

(10) $$\begin{array}{ll} \Gamma_{D_{1}\to x_{1}}^{AF} & =\frac{G^{2}P_{S}{∥h_{1}∥}^{2}{∥h_{SR}∥}^{2}\Xi_{1}}{G^{2}P_{S}{∥h_{1}∥}^{2}{∥h_{SR}∥}^{2}\Xi_{2}+{∥h_{1}∥}^{2}G^{2}N_{0}+N_{0}}\\ \; & ≃\frac{\phi {\bar{\gamma }}_{SR}{∥h_{1}∥}^{2}\Xi_{1}}{\phi {\bar{\gamma }}_{SR}{∥h_{1}∥}^{2}\Xi_{2}+{∥h_{1}∥}^{2}\phi +1}. \end{array}$$

According to the NOMA principle, the approximate SINR expressions for $D_{1}$ and $D_{2}$ are expressed as

(11) $$\Gamma_{D_{2}\to x_{1}}^{AF}≃\frac{\phi {\bar{\gamma }}_{SR}{∥h_{2}∥}^{2}\Xi_{1}}{\phi {\bar{\gamma }}_{SR}{∥h_{2}∥}^{2}\Xi_{2}+{∥h_{2}∥}^{2}\phi +1},$$

and

(12) $$\Gamma_{D_{2}\to x_{2}}^{AF}≃\frac{\phi {∥h_{2}∥}^{2}{\bar{\gamma }}_{SR}\Xi_{2}}{{∥h_{2}∥}^{2}\phi +1}.$$

## 3. Performance Analysis of NOMA-Enabled HSTRNs

In this section, we provide details of the step-by-step derivations of closed-form expressions of outage probabilities and ergodic capacities. Prior to that it is important to understand the different parameters of the wireless channel. The details of the channel models are given below:

### 3.1. Channel Model

First, we assume independent and identically distributed (i.i.d.) channel conditions for each hop. Moreover, under Shadowed-Rician fading model, the probability density function (PDF) of the squared amplitude of the channel coefficient $h_{SR}$ between satellite and the relay is given by [13]

(13) $$\begin{array}{ll} f_{{\left|h_{SR}\right|}^{2}}\left(x\right) & =\alpha {e^{-\beta x}}_{1}F_{1}\left(m_{SR};1;\delta x\right),x>0\\ \; & =\alpha \sum_{k=0}^{m_{SR}-1}\zeta \left(k\right)x^{k}e^{-\left(\beta -\delta \right)x}, \end{array}$$

where $\alpha =(2b_{SR}m_{SR}/{\left(2b_{SR}m_{SR}+\Omega_{SR}\right)}^{m_{SR}}/2b_{SR}$, $\beta =0.5b_{SR}$, $\delta =\Omega_{SR}/\left(2b_{SR}\right)\left(2b_{SR}m_{SR}+\Omega_{SR}\right)$ with $\Omega_{SR}$, $2b_{SR}$ and $m_{SR}$ denotes the average power, multipath components and the fading severity parameter, respectively. $\zeta \left(k\right)=\frac{{\left(-1\right)}^{k}{\left(1-m_{SR}\right)}_{k}\delta^{k}}{{\left(k!\right)}^{2}}$, and ${\left(•\right)}_{k}$ is the Pochhammer symbol. Moreover, the PDF of ${\bar{\gamma }}_{SR}$ is given as

(14) $$f_{{\bar{\gamma }}_{SR}}\left(x\right)=\overset{m_{SR}-1}{\underset{i_{1}=0}{\sum }}\cdots \overset{m_{SR}-1}{\underset{i_{M}=0}{\sum }}\frac{\Theta \left(M\right)}{{\left(\rho_{S}\right)}^{\Delta }}x^{\Delta -1}e^{-\left(\frac{\beta -\delta }{\rho_{S}}\right)x},$$

where $\Theta \left(M\right)=\alpha_{M}\prod_{\ell =1}^{M}\zeta \left(i_{\ell }\right)\prod_{j=1}^{M-1}ß(\sum_{l=1}^{j}i_{l}+j,i_{j+1}$+1), $\Delta =\sum_{q=1}^{M}i_{q}+M$ and ß(.,.) is the Beta function. The cumulative distribution function (CDF) of ${\bar{\Gamma }}_{SR}$ is expressed as

(15) $$F_{{\bar{\gamma }}_{SR}}\left(x\right)=1-\overset{m_{SR}-1}{\underset{i_{1}=0}{\sum }}\cdots \overset{m_{SR}-1}{\underset{i_{M}=0}{\sum }}\frac{\Theta \left(M\right)}{{\left(\rho_{S}\right)}^{\Delta }}\overset{\Delta -1}{\underset{p=0}{\sum }}\frac{\left(\Delta -1\right)!}{p!}{\left(\frac{\beta -\delta }{\rho_{S}}\right)}^{-\Delta +p}x^{p}e^{-\left(\frac{\beta -\delta }{\rho_{S}}\right)x}.$$

Regarding channels in ground, the PDF and CDF of ${∥h_{1}∥}^{2}$ are given as respectively

(16) $$f_{{∥h_{i}∥}^{2}}\left(x\right)=\frac{x^{N_{i}m_{i}-1}}{\Gamma \left(N_{i}m_{j}\right)\Lambda_{i}^{N_{i}m_{j}}}e^{-\frac{x}{\Lambda_{j}}},$$

(17) $$F_{{∥h_{i}∥}^{2}}\left(x\right)=1-e^{-\frac{x}{\Lambda_{i}}}\overset{N_{i}m_{i}-1}{\underset{n_{i}=0}{\sum }}\frac{x^{n_{i}}}{\Lambda_{i}^{n_{i}}n_{i}!}.$$

### 3.2. Outage Probability

Outage performance is defined as the maximum rate that can be guaranteed for considered system. Outage probability is one of the critical metrics for determining the performance of emergency services [16]. In other words, the minimum outage probability is closely related to the capacity. Therefore, it is important to characterize the outage behavior of the energy harvesting HSTRNs.

#### 3.2.1. DF Protocol

Let us denote $\Psi_{i}=2^{\frac{2R_{i}}{1-\chi }}-1$ as the threshold SNRs of *i*-th user to decode $x_{i}$. Then, the outage probability of $D_{1}$ is determined by

(18) $$\begin{array}{ll} P_{out,D_{1}}^{DF} & =\mathrm{Pr}\left(\min\left(\Gamma_{R\to x_{1}}^{DF},\Gamma_{D_{1}\to x_{1}}^{DF},\Gamma_{D_{2}\to x_{1}}^{DF}\right)<\Psi_{1}\right)\\ \; & =1-\mathrm{Pr}\left(\Gamma_{R\to x_{1}}^{DF}>\Psi_{1},\Gamma_{D_{1}\to x_{1}}^{DF}>\Psi_{1},\Gamma_{D_{2}\to x_{1}}^{DF}>\Psi_{1}\right). \end{array}$$

**Proposition** **1.** *The closed-form outage probability of signal $D_{1}$ can be expressed by*

(19) $$\begin{array}{ll} P_{out,D_{1}}^{DF} & =1-\sum_{i_{1}=0}^{m_{SR}-1}\cdots \sum_{i_{M}=0}^{m_{SR}-1}\Theta \left(M\right)\sum_{n_{1}=0}^{m_{1}N_{1}-1}\sum_{n_{2}=0}^{m_{2}N_{2}-1}\frac{{\left(\beta -\delta \right)}^{n_{1}+n_{2}-\Delta }}{n_{1}!n_{2}!\Lambda_{1}^{n_{1}}\Lambda_{2}^{n_{2}}}{\left(\frac{\kappa_{1}}{\rho_{S}\phi }\right)}^{n_{1}+n_{2}}\\ \; & \times \Gamma \left(\Delta -n_{1}-n_{2},\frac{\kappa_{1}\left(\beta -\delta \right)}{\rho_{S}},\frac{\left(\Lambda_{1}+\Lambda_{2}\right)\left(\beta -\delta \right)\kappa_{1}}{\Lambda_{1}\Lambda_{2}\phi \rho_{S}}\right). \end{array}$$

**Proof.**  Please refer to Appendix A for detailed proof of derivation of Equation (19).  □

Next, we derive the outage probability of $D_{2}$ which is given by

(20) $$\begin{array}{ll} P_{out,D_{2}}^{DF} & =\mathrm{Pr}\left(\min\left(\Gamma_{R\to x_{2}}^{DF},\Gamma_{D_{2}\to x_{2}}^{DF}\right)<\Psi_{2}\right)\\ \; & =1-\mathrm{Pr}\left(\Gamma_{R\to x_{2}}^{DF}>\Psi_{2},\Gamma_{D_{2}\to x_{2}}^{DF}>\Psi_{2}\right). \end{array}$$

Replacing Equations (2) and (8) into Equation (20), we get

(21) $$P_{out,D_{2}}^{DF}=1-\mathrm{Pr}\left({\bar{\gamma }}_{SR}>\kappa_{2},{∥h_{2}∥}^{2}>\frac{\kappa_{1}}{{\bar{\gamma }}_{SR}\phi }\right),$$

where $\kappa_{2}=\frac{\Psi_{2}}{\Xi_{2}}$. Following the approach of Appendix A, the closed-form outage probability of $D_{2}$ can, thus, be obtained as

(22) $$P_{out,D_{2}}^{DF}=1-\overset{m_{SR}-1}{\underset{i_{1}=0}{\sum }}\cdots \overset{m_{SR}-1}{\underset{i_{M}=0}{\sum }}\Theta \left(M\right)\overset{m_{2}N_{2}-1}{\underset{n_{2}=0}{\sum }}\frac{{\left(\beta -\delta \right)}^{n_{2}-\Delta }}{n_{2}!}{\left(\frac{\kappa_{2}}{\Lambda_{2}\rho_{S}\phi }\right)}^{n_{2}}\Gamma \left(\Delta -n_{2},\frac{\kappa_{2}\left(\beta -\delta \right)}{\rho_{S}},\frac{\kappa_{2}\left(\beta -\delta \right)}{\Lambda_{2}\phi \rho_{S}}\right).$$

#### 3.2.2. AF Protocol

The outage probability of $D_{1}$ can be written as

(23) $$P_{out,D_{1}}^{AF}=\mathrm{Pr}\left(\Gamma_{D_{1}\to x_{1}}^{AF}<\Psi_{1}\right).$$

**Proposition** **2.** *The closed-form expression of outage probability of $D_{1}$, in case of AF relaying, can be expressed as*

(24) $$\begin{array}{ll} P_{out,D_{1}}^{AF} & =1-2\sum_{i_{1}=0}^{m_{SR}-1}\cdots \sum_{i_{M}=0}^{m_{SR}-1}\Theta \left(M\right)\sum_{n_{1}=0}^{N_{1}m_{1}-1}\sum_{q=0}^{\Delta -1}\left(\begin{array}{c} \Delta -1\\ q \end{array}\right)\frac{{\left(\Lambda_{1}\phi \right)}^{q}}{n_{1}!{\left(\beta -\delta \right)}^{\Delta }}\\ \; & \times {\left(\frac{\kappa_{1}\left(\beta -\delta \right)}{\Lambda_{1}\phi \rho_{S}}\right)}^{\frac{\Delta +q+n_{1}}{2}}e^{-\frac{\kappa_{1}\left(\beta -\delta \right)}{\rho_{S}}}K_{\Delta -q-n_{1}}\left(2\sqrt{\frac{\kappa_{1}\left(\beta -\delta \right)}{\Lambda_{1}\phi \rho_{S}}}\right). \end{array}$$

**Proof.**  Please refer to Appendix B for detailed proof of derivation. □

Next, the outage probability of $D_{2}$ can be expressed as

(25) $$P_{out,D_{2}}^{AF}=\mathrm{Pr}\left(\Gamma_{D_{2}\to x_{2}}^{AF}<\Psi_{2}\right).$$

Then, $P_{out,D_{2}}^{AF}$ is rewritten by

(26) $$P_{out,D_{2}}^{AF}=1-\mathrm{Pr}\left({\bar{\gamma }}_{SR}>\frac{\kappa_{2}\left({∥h_{2}∥}^{2}\phi +1\right)}{\phi {∥h_{2}∥}^{2}}\right).$$

Following the similar steps indicated in Appendix B, the closed-form expression of outage probability of $D_{2}$, in case of AF relaying, can be formulated by

(27) $$\begin{array}{ll} P_{out,D_{2}}^{AF} & =1-2\sum_{i_{1}=0}^{m_{SR}-1}\cdots \sum_{i_{M}=0}^{m_{SR}-1}\Theta \left(M\right)\sum_{n_{2}=0}^{N_{2}m_{2}-1}\sum_{q=0}^{\Delta -1}\left(\begin{array}{c} \Delta -1\\ q \end{array}\right)\frac{{\left(\Lambda_{2}\phi \right)}^{q}}{n_{1}!{\left(\beta -\delta \right)}^{\Delta }}\\ \; & \times {\left(\frac{\theta_{2}\left(\beta -\delta \right)}{\Lambda_{2}\phi \rho_{S}}\right)}^{\frac{\Delta +q+n_{2}}{2}}e^{-\frac{\theta_{2}\left(\beta -\delta \right)}{\rho_{S}}}K_{\Delta -q-n_{2}}\left(2\sqrt{\frac{\theta_{2}\left(\beta -\delta \right)}{\Lambda_{2}\phi \rho_{S}}}\right). \end{array}$$

### 3.3. Ergodic Capacity

We now provide detailed derivations of ergodic capacity for DF and AF relaying protocols. It is worth mentioning that ergodic capacity of a wireless network is an important performance metric to determine the average link capacity.

#### 3.3.1. DF Protocol

Let us now derive the ergodic capacity for relay employing DF protocol. The achievable capacity at the $D_{1}$ can be computed by

(28) $$\begin{array}{ll} C_{D_{1}}^{DF} & =\frac{1-\chi }{2}\log_{2}\left(1+\min\left(\Gamma_{R\to x_{1}}^{DF},\Gamma_{D_{1}\to x_{1}}^{DF}\right)\right)\\ \; & =\frac{1-\chi }{2}\log_{2}\left(1+\min\left(\frac{P_{S}{∥h_{SR}∥}_{F}^{2}\Xi_{1}}{P_{S}{∥h_{SR}∥}_{F}^{2}\Xi_{1}+N_{0}},\frac{P_{R}{∥h_{1}∥}^{2}\Xi_{1}}{P_{R}{∥h_{1}∥}^{2}\Xi_{2}+N_{0}}\right)\right). \end{array}$$

In practice, the amount of harvested energy at the relay is always small, hence the transmit power of relay is much lower than that of the source. Thus, it is reasonable to assume that the SINR at the destinations is lower than the SINR at the relay, i.e., $\frac{P_{S}{∥h_{SR}∥}_{F}^{2}\Xi_{1}}{P_{S}{∥h_{SR}∥}_{F}^{2}\Xi_{1}+N_{0}}>\frac{P_{R}{∥h_{1}∥}^{2}\Xi_{1}}{P_{R}{∥h_{1}∥}^{2}\Xi_{2}+N_{0}}$. Hence, Equation (28) can be rewritten as

(29) $$\begin{array}{ll} C_{D_{1}}^{DF} & =\frac{1-\chi }{2}\log_{2}\left(1+\frac{\phi {\bar{\gamma }}_{SR}{∥h_{1}∥}^{2}\Xi_{1}}{\phi {\bar{\gamma }}_{SR}{∥h_{1}∥}^{2}\Xi_{2}+1}\right)\\ \; & =\frac{1-\chi }{2}\log_{2}\left(\frac{\phi {\bar{\gamma }}_{SR}{∥h_{1}∥}^{2}+1}{\phi {\bar{\gamma }}_{SR}{∥h_{1}∥}^{2}\Xi_{2}+1}\right). \end{array}$$

With the instantaneous capacity is derived in Equation (29), the ergodic capacity for user $D_{1}$ can be obtained as

(30) $$\begin{array}{ll} {\bar{C}}_{D_{1}}^{DF} & =E\left\{\frac{1-\chi }{2}\log_{2}\left(\phi {\bar{\gamma }}_{SR}{∥h_{1}∥}^{2}+1\right)\right\}\\ \; & -E\left\{\frac{1-\chi }{2}\log_{2}\left(\phi {\bar{\gamma }}_{SR}{∥h_{1}∥}^{2}\Xi_{2}+1\right)\right\}\\ & =\underset{L_{1}}{\underbrace{\frac{1-\chi }{2\ln2}\int_{0}^{\infty }\frac{1-F_{H_{1}}\left(x\right)}{1+x}dx}}-\underset{L_{2}}{\underbrace{\frac{1-\chi }{2}\int_{0}^{\infty }\frac{1-F_{H_{2}}\left(x\right)}{1+x}dx}}, \end{array}$$

where $H_{1}=\phi {\bar{\gamma }}_{SR}{∥h_{1}∥}^{2}$, $H_{2}=\phi {\bar{\gamma }}_{SR}{∥h_{1}∥}^{2}\Xi_{2}$. Based on result from [3.471.9] in [34], Equation (17) and after some algebraic manipulations, $F_{P_{1}}\left(x\right)$ can be obtained as

(31) $$\begin{array}{ll} F_{H_{1}}\left(x\right) & =1-\sum_{i_{1}=0}^{m_{SR}-1}\cdots \sum_{i_{M}=0}^{m_{SR}-1}\Theta \left(M\right)\sum_{p=0}^{\Delta -1}\frac{\left(\Delta -1\right)!2^{-N_{1}m_{1}-p+1}}{p!\Gamma \left(N_{1}m_{1}\right){\left(\beta -\delta \right)}^{\Delta }}\\ \; & \times {\left(\sqrt{\frac{4x\left(\beta -\delta \right)}{\rho_{S}\phi \Lambda_{1}}}\right)}^{N_{1}m_{1}+p}K_{N_{1}m_{1}-p}\left(\sqrt{\frac{4x\left(\beta -\delta \right)}{\rho_{S}\phi \Lambda_{1}}}\right). \end{array}$$

With the help of [Eq. 9.34.3 and Eq. 7.811.5] in [34] and after some variable substitutions and manipulations, $J_{1}$ and $J_{2}$ can be written as

(32) $$L_{1}=\frac{1-\chi }{2\ln2}\overset{m_{SR}-1}{\underset{i_{1}=0}{\sum }}\cdots \overset{m_{SR}-1}{\underset{i_{M}=0}{\sum }}\Theta \left(M\right)\overset{\Delta -1}{\underset{p=0}{\sum }}\frac{\Gamma \left(\Delta \right)}{\Gamma \left(N_{1}m_{1}\right)p!{\left(\beta -\delta \right)}^{\Delta }}G_{1,3}^{3,1}\left(\frac{\beta -\delta }{\Lambda_{1}\phi \rho_{S}}\left|\begin{array}{c} 0\\ 0,N_{1}m_{1},p \end{array}\right.\right).$$

Similarly, $L_{2}$ can be calculated as follows

(33) $$L_{2}=\frac{1-\chi }{2\ln2}\overset{m_{SR}-1}{\underset{i_{1}=0}{\sum }}\cdots \overset{m_{SR}-1}{\underset{i_{M}=0}{\sum }}\Theta \left(M\right)\overset{\Delta -1}{\underset{p=0}{\sum }}\frac{\Gamma \left(\Delta \right)}{\Gamma \left(N_{1}m_{1}\right)p!{\left(\beta -\delta \right)}^{\Delta }}G_{1,3}^{3,1}\left(\frac{\beta -\delta }{\Lambda_{1}\phi \Xi_{2}\rho_{S}}\left|\begin{array}{c} 0\\ 0,N_{1}m_{1},p \end{array}\right.\right).$$

With the help of Equations (32) and (33), we obtain the achievable capacity of $D_{1}$. Next, the achievable capacity at the $D_{2}$ is given as

(34) $$C_{D_{2}}^{DF}=\frac{1-\chi }{2}\log_{2}\left(1+\min\left(\Gamma_{R\to x_{2}}^{DF},\Gamma_{D_{2}\to x_{2}}^{DF}\right)\right)$$

Similarly, the ergodic capacity of the $D_{2}$ is given as

(35) $${\bar{C}}_{D_{2}}^{DF}=\frac{1-\chi }{2\ln2}\overset{m_{SR}-1}{\underset{i_{1}=0}{\sum }}\cdots \overset{m_{SR}-1}{\underset{i_{M}=0}{\sum }}\Theta \left(M\right)\overset{\Delta -1}{\underset{p=0}{\sum }}\frac{\Gamma \left(\Delta \right)}{\Gamma \left(N_{2}m_{2}\right)p!{\left(\beta -\delta \right)}^{\Delta }}G_{1,3}^{3,1}\left(\frac{\beta -\delta }{\Lambda_{2}\phi \Xi_{2}\rho_{S}}\left|\begin{array}{c} 0\\ 0,N_{2}m_{2},p \end{array}\right.\right).\;$$

#### 3.3.2. AF Protocol

The achievable capacity at the $D_{i}$ in case of AF relaying can be calculated by

(36) $$C_{D_{i}}^{AF}=\frac{1-\chi }{2}\log_{2}\left(1+\Gamma_{D_{i}\to x_{i}}^{AF}\right).$$

Moreover, the ergodic capacity of user $D_{1}$ can be formulated as

(37) $$\begin{array}{c} {\bar{C}}_{D_{1}}^{AF}=\frac{1-\chi }{2\ln2}\overset{\Xi_{2}/\Xi_{1}}{\underset{0}{\int }}\frac{1-F_{\Gamma_{D_{1}\to x_{1}}}\left(x_{1}\right)}{1+x_{1}}dx_{1}. \end{array}$$

In the above expression, the CDF of $\Gamma_{D_{1}\to x_{1}}$ can be expressed as

(38) $$\begin{array}{ll} F_{\Gamma_{D_{1}\to x_{1}}}\left(x_{1}\right) & =1-2\sum_{i_{1}=0}^{m_{SR}-1}\cdots \sum_{i_{M}=0}^{m_{SR}-1}\Theta \left(M\right)\sum_{n_{1}=0}^{N_{1}m_{1}-1}\sum_{q=0}^{\Delta -1}\left(\begin{array}{c} \Delta -1\\ q \end{array}\right)\frac{{\left(\Lambda_{1}\phi \right)}^{q}}{n_{1}!{\left(\beta -\delta \right)}^{\Delta }}\\ \; & \times {\left(\frac{x_{1}\left(\beta -\delta \right)}{\left(\Xi_{1}-\Xi_{2}x_{1}\right)\Lambda_{1}\phi \rho_{S}}\right)}^{\frac{\Delta +q+n_{1}}{2}}e^{-\frac{x_{1}\left(\beta -\delta \right)}{\left(\Xi_{1}-\Xi_{2}x_{1}\right)\rho_{S}}}K_{\Delta -q-n_{1}}\left(\frac{x_{1}\left(\beta -\delta \right)}{\left(\Xi_{1}-\Xi_{2}x_{1}\right)\Lambda_{1}\phi \rho_{S}}\right). \end{array}$$

Now, using Gaussian-Chebyshev with $\phi_{n}=\cos\left(\frac{2n-1}{2N}\pi \right)$. After solving the integral, we can obtain ${\bar{C}}_{D_{1}}^{AF}$ as

(39) $$\begin{array}{ll} {\bar{C}}_{D_{1}}^{AF} & \approx \frac{1-\chi }{\ln2}\sum_{i_{1}=0}^{m_{SR}-1}\cdots \sum_{i_{M}=0}^{m_{SR}-1}\Theta \left(M\right)\sum_{n_{1}=0}^{N_{1}m_{1}-1}\sum_{q=0}^{\Delta -1}\left(\begin{array}{c} \Delta -1\\ q \end{array}\right)\frac{{\left(\Lambda_{1}\phi \right)}^{q}}{n_{1}!{\left(\beta -\delta \right)}^{\Delta }}\frac{\pi }{N}\sum_{n=1}^{N}\frac{\Xi_{1}\sqrt{1-\phi_{n}^{2}}}{2-\Xi_{1}\left(1-\phi_{n}\right)}\\ \; & \times e^{-\frac{\left(1+t\right)}{\Xi_{2}\rho_{S}\left(1-t\right)}\left(\beta -\delta \right)}{\left(\frac{\left(\beta -\delta \right)\left(1+\phi_{n}\right)}{\Lambda_{1}\phi \rho_{S}\Xi_{2}\left(1-\phi_{n}\right)}\right)}^{\frac{\Delta +q+n_{1}}{2}}K_{\Delta -q-n_{1}}\left(2\sqrt{\frac{\left(\beta -\delta \right)\left(1+\phi_{n}\right)}{\Lambda_{1}\phi \rho_{S}\Xi_{2}\left(1-\phi_{n}\right)}}\right). \end{array}$$

In a similar manner, the ergodic of $D_{2}$ can be expressed as

(40) $${\bar{C}}_{D_{2}}^{AF}=\frac{1-\chi }{2\ln2}\overset{\infty }{\underset{0}{\int }}\frac{1-F_{\Gamma_{\Gamma_{D_{2}\to x_{2}}}}\left(x_{2}\right)}{1+x_{2}}dx_{2},$$

where $F_{\Gamma_{\Gamma_{D_{2}\to x_{2}}}}$ can be expressed as

(41) $$\begin{array}{ll} F_{\Gamma_{\Gamma_{D_{2}\to x_{2}}}}\left(x_{2}\right) & =1-\sum_{i_{1}=0}^{m_{SR}-1}\cdots \sum_{i_{M}=0}^{m_{SR}-1}\Theta \left(M\right)\sum_{n_{1}=0}^{N_{1}m_{1}-1}\sum_{q=0}^{\Delta -1}\left(\begin{array}{c} \Delta -1\\ q \end{array}\right)\frac{{\left(\Lambda_{1}\phi \right)}^{q}e^{-\frac{x_{2}\left(\beta -\delta \right)}{\Xi_{2}\rho_{S}}}}{n_{1}!{\left(\beta -\delta \right)}^{\Delta }2^{\Delta +q+n_{1}-1}}\\ \; & \times {\left(\sqrt{\frac{4x_{2}\left(\beta -\delta \right)}{\Xi_{2}\Lambda_{1}\phi \rho_{S}}}\right)}^{\Delta +q+n_{1}}K_{\Delta -q-n_{1}}\left(\sqrt{\frac{4x_{2}\left(\beta -\delta \right)}{\Xi_{2}\Lambda_{1}\phi \rho_{S}}}\right). \end{array}$$

Submitting Equation (41) into Equation (40), we get

(42) $$\begin{array}{ll} {\bar{C}}_{D_{2}}^{AF} & =\frac{1-\chi }{\ln2}\sum_{i_{1}=0}^{m_{SR}-1}\cdots \sum_{i_{M}=0}^{m_{SR}-1}\Theta \left(M\right)\sum_{n_{1}=0}^{N_{1}m_{1}-1}\sum_{q=0}^{\Delta -1}\left(\begin{array}{c} \Delta -1\\ q \end{array}\right)\frac{{\left(\Lambda_{1}\phi \right)}^{q}}{n_{1}!{\left(\beta -\delta \right)}^{\Delta }2^{\Delta +q+n_{1}}}\\ \; & \times \int_{0}^{\infty }\frac{e^{-\frac{x_{2}\left(\beta -\delta \right)}{\Xi_{2}\rho_{S}}}}{1+x_{2}}{\left(\sqrt{\frac{4x_{2}\left(\beta -\delta \right)}{\Xi_{2}\Lambda_{1}\phi \rho_{S}}}\right)}^{\Delta +q+n_{1}}K_{\Delta -q-n_{1}}\left(\sqrt{\frac{4x_{2}\left(\beta -\delta \right)}{\Xi_{2}\Lambda_{1}\phi \rho_{S}}}\right)dx_{2}. \end{array}$$

With the help of ${\left(1+x_{2}\right)}^{-1}=G_{1,1}^{1,1}\left(x_{2}\left|\begin{array}{c} 0\\ 0 \end{array}\right.\right)$ and [9.34.3] in [34], we can rewrite ${\bar{C}}_{D_{2}}^{AF}$ as

(43) $$\begin{array}{ll} {\bar{C}}_{D_{2}}^{AF} & =\frac{1-\chi }{2\ln2}\sum_{i_{1}=0}^{m_{SR}-1}\cdots \sum_{i_{M}=0}^{m_{SR}-1}\Theta \left(M\right)\sum_{n_{1}=0}^{N_{1}m_{1}-1}\sum_{q=0}^{\Delta -1}\left(\begin{array}{c} \Delta -1\\ q \end{array}\right)\frac{{\left(\Lambda_{1}\phi \right)}^{q}}{n_{1}!{\left(\beta -\delta \right)}^{\Delta }}\\ \; & \times \int_{0}^{\infty }e^{-\frac{\left(\beta -\delta \right)}{\Xi_{2}\rho_{S}}x_{2}}G_{1,1}^{1,1}\left(x_{2}\left|\begin{array}{c} 0\\ 0 \end{array}\right.\right)G_{0,2}^{2,0}\left(\frac{\left(\beta -\delta \right)}{\Xi_{2}\Lambda_{1}\phi \rho_{S}}x_{2}\left|\begin{array}{c} -\\ \Delta ,q+n_{1} \end{array}\right.\right)dx_{2}. \end{array}$$

Based on [Eq 2.6.2] in [35], the closed-form expression for ergodic capacity of $D_{2}$ is given by

(44) $$\begin{array}{ll} {\bar{C}}_{D_{2}}^{AF} & =\frac{1-\chi }{2\ln2}\sum_{i_{1}=0}^{m_{SR}-1}\cdots \sum_{i_{M}=0}^{m_{SR}-1}\Theta \left(M\right)\sum_{n_{1}=0}^{N_{1}m_{1}-1}\sum_{q=0}^{\Delta -1}\left(\begin{array}{c} \Delta -1\\ q \end{array}\right)\frac{{\left(\Lambda_{1}\phi \right)}^{q}}{n_{1}!{\left(\beta -\delta \right)}^{\Delta }}\\ \; & \times G_{1,[1:0],0,[1:2]}^{1,1,0,1,2}\left(\left.\begin{array}{c} \frac{\Xi_{2}\rho_{S}}{\left(\beta -\delta \right)}\\ \frac{1}{\Lambda_{1}\varphi } \end{array}\right.\begin{array}{c} 1\\ 0;-\\ -\\ 0;\Delta ,q+n_{1} \end{array}\right). \end{array}$$

## 4. Numerical and Simulation Results

This section provides the numerical results along with the relevant discussion. To verify the accuracy of the expressions, we compare the analytical results with Monte Carlo simulation results and the parameters for numerical results can be seen in Table 2.

**Figure 2:**
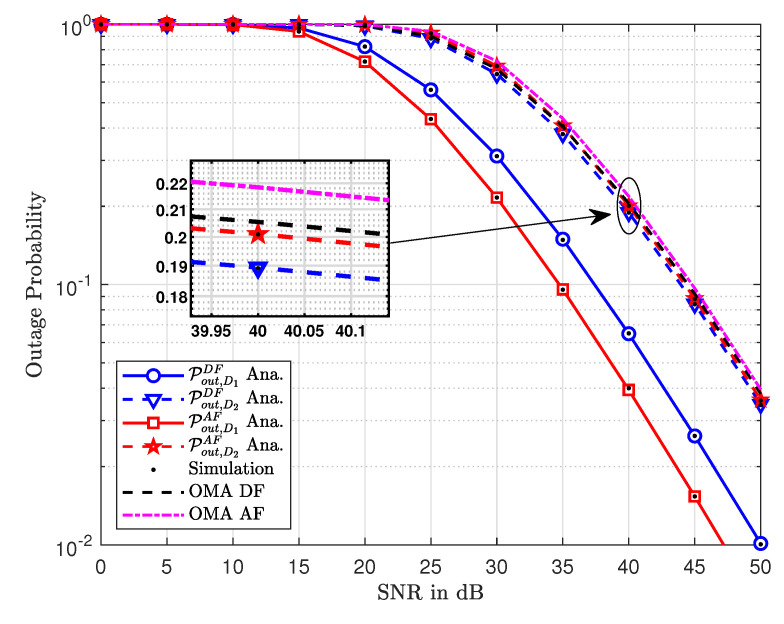
The outage probability versus transmit SNR.

Figure 2 shows the outage probability as a function of increasing values of SNR. In general, one can note that the outage probability decreases with an increase in the values of SNR. This is true for both the NOMA and OMA schemes. As expected, for the same values of SNR, the outage probability of DF relays is less than that of AF relays. This is independent of multiple access techniques. However, NOMA outperforms OMA for both DF and AF relaying. Additionally, the simulation results closely follow the analytical curves which indicate the accuracy of the derived expressions.

**Figure 3:**
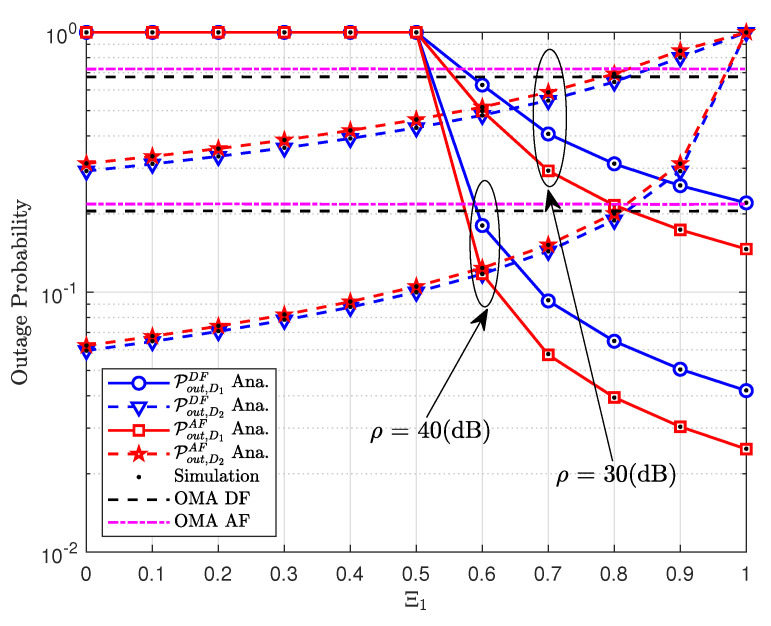
The outage probability versus the power allocation coefficients $\Xi_{1}$.

Figure 3 indicates the impact of power allocation coefficients $\Xi_{1}$ on the outage performance. The lowest outage probability can be observed in the range of $\Xi_{1}$ from 0.5 to 1 for case of users $D_{1}$ and $D_{2}$ in AF mode. It is worth noting that the performance gap among two users in NOMA scheme is resulted from different power allocation coefficients while outage probability remains unchanged in OMA scheme.

**Figure 4:**
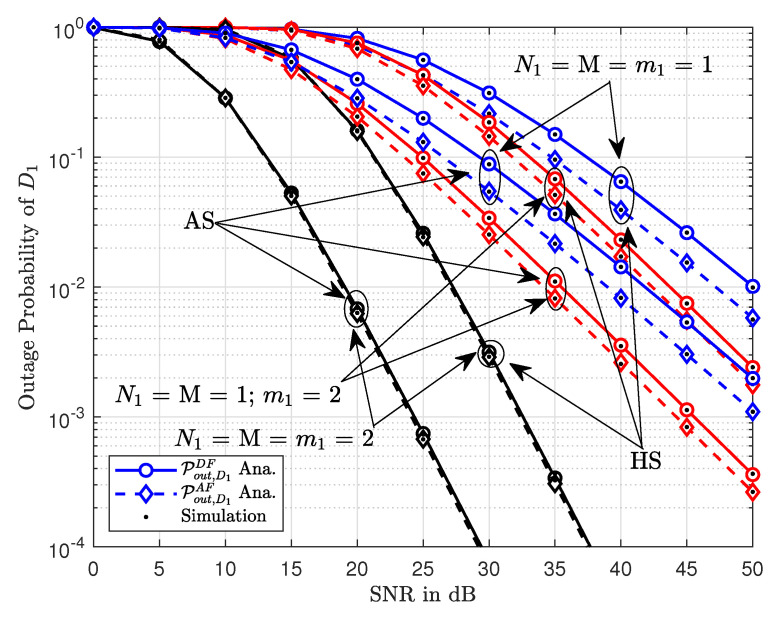
The outage probability of $D_{1}$ versus transmit SNR.

Figure 4 shows the outage probability of $D_{1}$ as the function of transmitting SNR. It can be seen that the outage probability decreases with an increase in transmitting SNR. The comparison is provided between the outage probabilities of AS and HS communication conditions. Please note that the outage probability of HS is significantly higher than the outage probability of AS. This is because the outage probability is hampered by the communication conditions between satellite and relay. It can also be noted that the difference between the outage probabilities of AF and DF relays is reduced for larger values of Nakagami-*m* factors.

**Figure 5:**
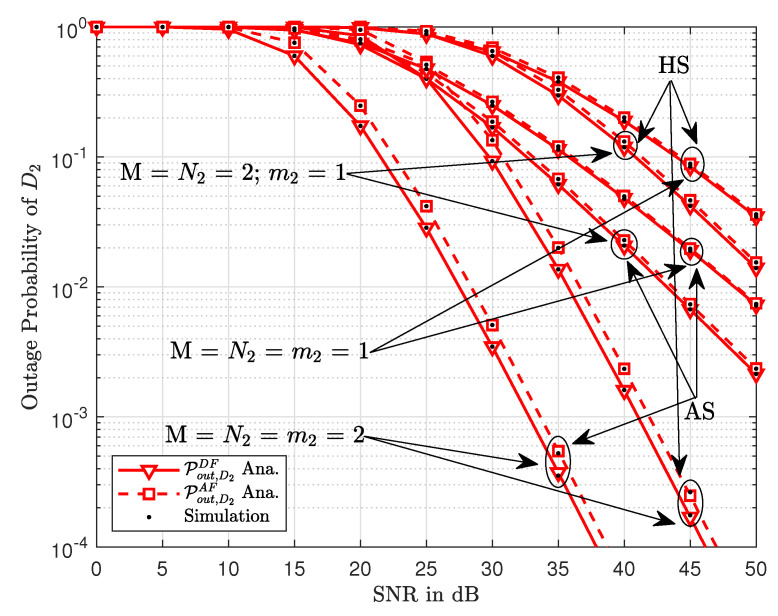
The outage probability of $D_{2}$ versus transmit SNR.

Similar trends can be observed in Figure 5 where the outage probability of $D_{2}$ is plotted against the transmitted SNR. However, we note that the Nakagami-*m* factor has a completely different impact on the outage probabilities in this scenario. As opposed to Figure 4, the outage probability curves of AF and DF in Figure 5 are closer to each other at lower values of the Nakagami-*m* factor. This shows that the larger values of *m* have more impact on the outage performance of AF and DF relays.

**Figure 6:**
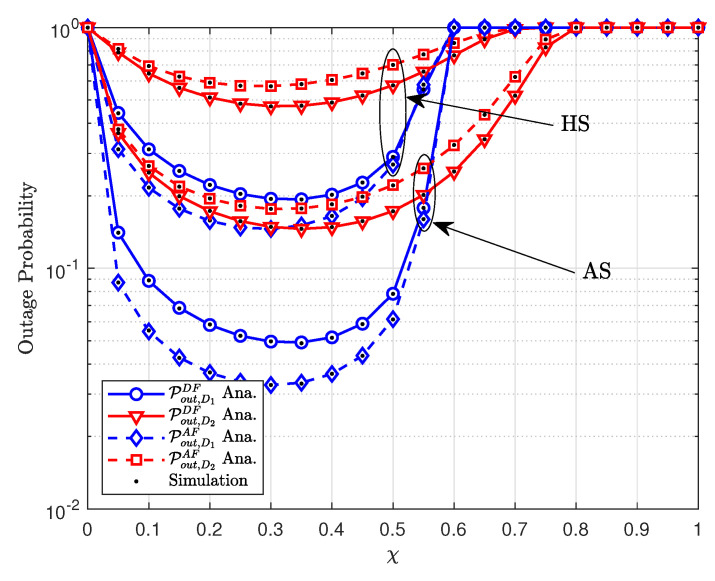
The outage probability versus transmit χ.

Figure 6 shows the outage probability against the power-splitting factor of χ. Please note that larger values of χ indicate that the more power is reserved for information processing while the rest is reserved for energy harvesting. One can observe that with an increase in the values of χ, the outage probability first decreases and then increases illustrating a convex trend. This is mainly due to the functionality of the terrestrial relay. Initially, more power is reserved at relay for energy harvesting, and, around χ=0.3, the outage probability reaches the lowest value. As the value of χ increases, more power is reserved for information processing while no power is left for energy harvesting which increases the outage probability. Furthermore, when the satellite to the relay channel is relatively good, the dip in outage probability is more prominent. By contrast, for the case of HS, the outage probability curves are higher which is due to the impact of shadows.

**Figure 7:**
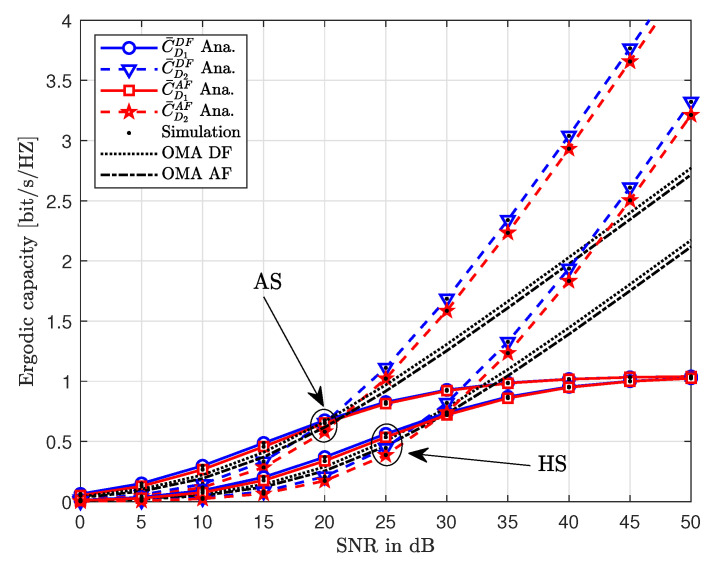
The ergodic capacity versus transmit SNR with different values of $R_{th}$, where ζ=0.01, ϑ=0.1, δ=0.1, η=0.9 and m=1.

To further investigate the performance of NOMA-enabled HSTRNs, Figure 7 demonstrates the ergodic capacity as a function of transmitting SNR. Generally, one can note that an increase in the value SNR improves the ergodic capacity of the users. At lower values of SNR around 5 dB, the outage probabilities for HS and AS are almost the same. However, as the SNR increases, the ergodic capacity for the case of AS reaches higher values than the ergodic capacity of HS. This shows that the shadowing has a greater effect at larger values of transmitting SNR. Furthermore, one can also note that the ergodic capacity curves of $D_{2}$ continue to increase with an increase in SNR. On the contrary, the ergodic capacity curves reach a ceiling due to the effect of interference as the SNR goes beyond 30 dB. This signifies that a large transmit SNR favors the ergodic capacity of $D_{2}$ more than the ergodic capacity of $D_{1}$ independent of relaying protocol.

**Figure 8:**
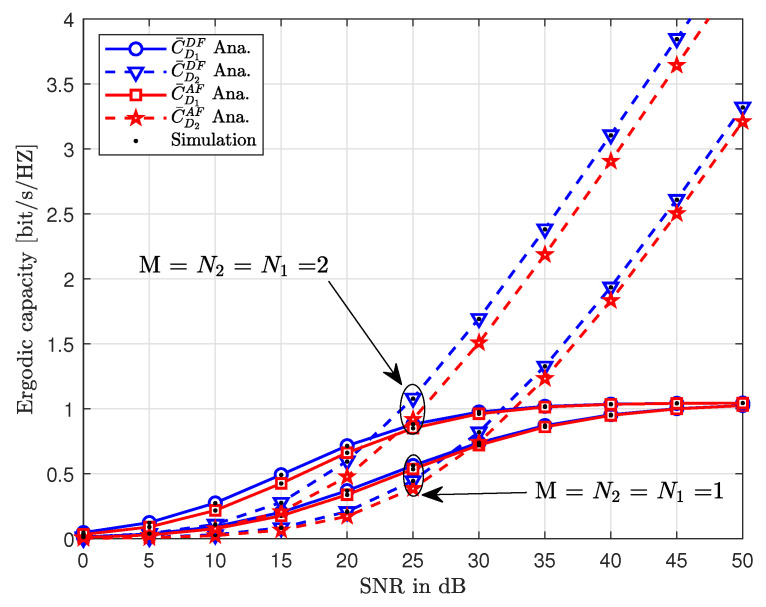
Ergodic capacity versus transmit SNR with different values of, where ζ=0.01, ϑ=0.1, δ=0.1, η=0.9 and m=1.

Figure 8 shows the ergodic capacity against the increasing values of SNR. Similar to Figure 7, we note that the ergodic capacity increases with increasing SNR in Figure 8. For the larger value of $N_{1}=N_{2}$, the gap between AF and DF curves is larger, whereas, this gap reduces at smaller values of $N_{1}=N_{2}$. Additionally, as opposed to Figure 7, the gap between $D_{1}$ and $D_{2}$ curves is more prominent at smaller values of SNR, wherein, $D_{1}$ outperforms $D_{2}$ in terms of ergodic capacity. However, the performance of $D_{1}$ is affected by the interference which results in a ceiling at higher values of SNR.

## 5. Discussion

In this paper, we derived the closed-form expressions of outage probabilities and ergodic capacities. Both AF and DF relaying protocols were considered to perform a comprehensive analysis. It was observed that the ergodic capacity curves of far users continue to increase with an increase in SNR, whereas, the ergodic capacity curves of near user reach a ceiling due to the effect of interference. Furthermore, it was unveiled that the outage probability of HS is significantly higher than the outage probability of AS because of the impact of communication conditions between satellite and relay.

This work can be extended in several ways. One of the exciting research directions, in this regard, can be the consideration of hardware impairments in the analysis. This would help unveil the impact of such impairments on network performance. Another important aspect to explore is relay selection, whereby the satellite could select the appropriate relay for transmission of messages. It is anticipated to further improve the performance of HSTRNs. These important yet challenging issues will be addressed in future studies.

## 6. Conclusions

The joint formation of space and terrestrial segments is essential for the provisioning of emergency services. This work, therefore, investigates the performance of NOMA-enabled HSTRNs with a wireless-powered terrestrial relay. More users can be served in the same time with NOMA. Different performance or different services can be seen in these users. To confirm these concerns, the expressions and results are provided in this paper as great significance for the performance evaluation of HSTRNs.

## Figures and Tables

**Table 1:** Key parameters of the system model.

Symbol	Description
$\Xi_{i}$	The power allocation coefficient i∈{1,2}
$P_{S}$	The transmit power at S
$P_{R}$	The transmit power at R
$n_{R}$	The AWGN with variance $N_{0}$
$n_{D_{i}}$	The AWGN with variance $N_{0}$
η	The energy conversion efficiency and $\eta \in \left(0,1\right]$
χ	The power splitting factor
*T*	The time duration
$R_{i}$	The target rate at $D_{i}$

**Table 2:** Table of parameters for numerical results.

Definition	Values
Monte Carlo simulations repeated	$10^{6}$ iterations
Power allocation coefficients	$\Xi_{1}=0.8$ and $\Xi_{2}=0.2$
Target rate	$R_{1}=0.5$ and $R_{2}=1$(BPCU) in which BPCU is short for bit per channel use.
The average shadowing (AS)	$\left(m_{SR}=5,b_{SR}=0.251,\Omega_{SR}=0.279\right)$
The heavy shadowing (HS)	$\left(m_{SR}=1,b_{SR}=0.063,\Omega_{SR}=0.0007\right)$
The energy conversion efficiency	η=0.9
The power splitting factor	χ=0.1
The factor and mean of $D_{i}$	$\Omega_{1}=\Omega_{2}=1$ and $m_{1}=m_{2}=1$
The antennas of satellite and $D_{i}$	M=1 and $N_{i}=1$

## Appendix A. Proof of Proposition 1

By using Equations (2), (7) and (8), the outage probability for the case of DF relay can be expressed as

(A1) $$P_{out,D_{1}}^{DF}=1-\mathrm{Pr}\left({\bar{\gamma }}_{SR}>\kappa_{1},{∥h_{1}∥}^{2}>\frac{\kappa_{1}}{\phi {\bar{\gamma }}_{SR}}\right),$$

where $\kappa_{1}=\frac{\Psi_{1}}{\Xi_{1}-\Psi_{1}\Xi_{2}}$. Based on result from Equation (15) and CDF of ${∥h_{i}∥}^{2}$, it can be rewritten as

(A2) $$\begin{array}{ll} P_{out,D_{1}}^{DF} & =1-\int_{\theta }^{\infty }f_{{\bar{\gamma }}_{SR}}\left(x\right){\bar{F}}_{{∥h_{1}∥}^{2}}\left(\frac{\kappa_{1}}{\phi x}\right)dx\\ \; & =1-\sum_{i_{1}=0}^{m_{SR}-1}\cdots \sum_{i_{M}=0}^{m_{SR}-1}\sum_{n_{1}=0}^{N_{1}m_{1}-1}\frac{\Theta \left(M\right)}{n_{1}!{\left(\rho_{S}\right)}^{\Delta }}{\left(\frac{\kappa_{1}}{\phi \Lambda_{1}}\right)}^{n_{1}}\\ \; & \times \int_{\theta }^{\infty }x^{\Delta -n_{1}-1}e^{-\frac{\kappa_{1}}{\phi \Lambda_{1}x}-\frac{\left(\beta -\delta \right)x}{\rho_{S}}}dx \end{array}$$

Using the results of [Eq.4] in [16] and after some straightforward mathematical steps, outage probability is expressed as the generalized incomplete Gamma function

(A3) $$\overset{\infty }{\underset{x}{\int }}t^{a-1}e^{-t-\frac{b}{t}}dt=\Gamma \left(a,x,b\right),$$

where Γ(a,x,b) is the generalized incomplete Gamma function [16]. Then, substituting Equation (A3) into Equation (A2), the expression in Equation (19) can be obtained.

## Appendix B. Proof of Proposition 2

The outage probability for AF relaying case is given as

(A4) $$\begin{array}{ll} P_{out,D_{1}}^{AF} & =1-\mathrm{Pr}\left({∥h_{1}∥}^{2}>\frac{\kappa_{1}}{\phi \left({\bar{\gamma }}_{SR}-\kappa_{1}\right)}\right)\\ \; & =1-\int_{\kappa_{1}}^{\infty }\left(1-F_{{∥h_{1}∥}^{2}}\left(\frac{\kappa_{1}}{\phi \left(x-\kappa_{1}\right)}\right)\right)f_{{\bar{\Gamma }}_{SR}}\left(x\right)dx \end{array}$$

After performing some simple transformations, we can rewrite the above expression as

(A5) $$\begin{array}{ll} P_{out,D_{1}}^{AF} & =1-\sum_{i_{1}=0}^{m_{SR}-1}\cdots \sum_{i_{M}=0}^{m_{SR}-1}\frac{\Theta \left(M\right)}{{\left(\rho_{S}\right)}^{\Delta }}\sum_{n_{1}=0}^{N_{1}m_{1}-1}{\frac{1}{n_{1}!}\left(\frac{\kappa_{1}}{\Lambda_{1}\phi }\right)}^{n_{1}}e^{-\frac{\kappa_{1}\left(\beta -\delta \right)}{\rho_{S}}}\\ \; & \times \int_{0}^{\infty }\frac{{\left(t+\kappa_{1}\right)}^{\Delta -1}}{t^{n_{1}}}e^{-\frac{\kappa_{1}}{\Lambda_{1}\phi t}}e^{-\left(\frac{\beta -\delta }{\rho_{S}}\right)t}dt \end{array}$$

Using the identity [1.111] in [34] and after some mathematical manipulations, we obtain

(A6) $$\begin{array}{ll} P_{out,D_{1}}^{AF} & =1-\sum_{i_{1}=0}^{m_{SR}-1}\cdots \sum_{i_{M}=0}^{m_{SR}-1}\frac{\Theta \left(M\right)}{{\left(\rho_{S}\right)}^{\Delta }}\sum_{n_{1}=0}^{N_{1}m_{1}-1}\sum_{q=0}^{\Delta -1}\left(\begin{array}{c} \Delta -1\\ q \end{array}\right)\frac{{\left(\kappa_{1}\right)}^{q}}{n_{1}!}{\left(\frac{\kappa_{1}}{\lambda_{1}\phi }\right)}^{n_{1}}\\ \; & \times \overset{\infty }{\underset{0}{\int }}e^{-\frac{\kappa_{1}\left(\beta -\delta \right)}{\rho_{S}}}t^{\Delta -q-n_{1}-1}e^{-\frac{\kappa_{1}}{\lambda_{1}\phi t}}e^{-\left(\frac{\beta -\delta }{\rho_{S}}\right)t}dt \end{array}$$

Now, with the help [3.471.9] in [34], we can obtain the closed-form expression for $D_{1}$.
