# Erratum: Do, D.-T., et al. Hybrid Satellite-Terrestrial Relay Network: Proposed Model and Application of Power Splitting Multiple Access. *Sensors* 2020, *20*, 4296

**DOI:** 10.3390/s21010186

**Published:** 2020-12-29

**Authors:** Dinh-Thuan Do, Anh-Tu Le, Rupak Kharel, Adão Silva, Mohammad Abu Shattal

**Affiliations:** 1 Department of Computer Science and Information Engineering, College of Information and Electrical Engineering, Asia University, Taichung City 41354, Taiwan; 2 Faculty of Electronics Technology, Industrial University of Ho Chi Minh City (IUH), Ho Chi Minh City 700000, Vietnam; leanhtu@iuh.edu.vn; 3 Department of Computing and Mathematics, Manchester Metropolitan University, Manchester M15 6BH, UK; 4 Instituto de Telecomunicações (IT) and Departamento de Eletrónica, Telecomunicações e Informática (DETI), University of Aveiro, 3810-193 Aveiro, Portugal; asilva@av.it.pt; 5 Department of Computer Science and Engineering, The Ohio State University, 2015 Neil Avenue, Columbus, OH 43212, USA; abushattal@ieee.org

The authors wish to make the following erratum to this paper [1].

Funding is incorrect and must be replaced by the following Funding:
